# Outcomes of severe peripartum cardiomyopathy and mechanical circulatory support: a case series

**DOI:** 10.1186/s40981-021-00484-2

**Published:** 2021-11-02

**Authors:** Yuki Kiriyama, Yuki Kinishi, Daisuke Hiramatu, Akinori Uchiyama, Yuji Fujino, Koichi Toda, Chiyo Ootaki

**Affiliations:** 1 Department of Anesthesiology and Intensive Care, Osaka University Graduate School of Medicine, 2-2 Yamadaoka, Suita City, Osaka, 565-0871 Japan; 2 Department of Cardiovascular Surgery, Osaka University Graduate School of Medicine, Osaka, Japan

**Keywords:** Cardiomyopathy, Heart failure, Pregnancy, Mechanical circulatory support, Bridge to transplantation, Bridge to recovery

## Abstract

**Background:**

We present three cases of severe peripartum cardiomyopathy (PPCM) that required mechanical circulatory supports.

**Case presentation:**

Case 1: A 33-year-old woman developed acute heart failure (AHF) after normal spontaneous delivery. Intra-aortic balloon pump (IABP) was inserted on postpartum day (PD) 10 with a peripartum cardiomyopathy (PPCM), which was withdrawn on PD 30 after medical treatment including anti-prolactin drugs.

Case 2: A 44-year-old woman developed AHF 1 month after vaginal delivery. IABP or extra-corporeal membrane oxygenation (ECMO) was not effective and a biventricular assist device was inserted. It was withdrawn on PD 85 after improvement of left ventricular ejection fraction (LVEF).

Case3: A 37-year-old woman was transferred with a diagnosis of PPCM. Cardiac function unimproved by IABP or ECMO, and a left ventricular assist device was implanted. It was withdrawn on PD 386 after recovery of LVEF.

**Conclusion:**

All the cases with PPCM recovered after mechanical circulatory supports and resumed social lives.

## Background

Peripartum cardiomyopathy (PPCM) is a heart failure secondary to reduced contractility due to unknown etiology during the peripartum period in previously healthy women. It is relatively rare with an incidence of one case per 10,000–20,000 deliveries [1]. Patients are generally treated for heart failure, and approximately half of them recover [2, 3]. However, patients who maintain cardiomegaly for 6 months or longer have an extremely poor prognosis [2].

There are several types of mechanical circulatory supports (MCSs) and are typically classified as either temporary or durable [4]. Temporary MCS devices include intra-aortic balloon pump (IABP), veno-arterial extracorporeal membrane oxygenation (VA-ECMO), and temporary ventricular assist devices (VAD). Durable MCS devices include implantable VADs that are used as destination therapy for end-stage cardiac failure. There are a few reports on the use of MCS for PPCM, and no studies on it appreciate induction timing of MCS or outcomes [5, 6]. Herein, we present three cases of severe PPCM that required MCS.

## Case presentation

Over the past 5 years, we encountered three patients with severe PPCM who were unresponsive to medical therapy and required MCS. The profile of the three cases who required MCSs are presented in Table 1, treatment details in Table 2, and cardiac data in Table 3.

**Table 1:** Profiles of 3 cases

	Case 1	Case 2	Case 3
Age of PPCM onset, years	33	44	37
BMI, kg/m^2^	18.9	24.5	28.4
Pregnancy history	Gravida 1, Para 1	Gravida 5, Para 5	Gravida 1, Para 1
Family history	None	None	Mother: DCM
Delivery	Vaginal	Vaginal	Vaginal
	Single-fetal	Single-fetal	Single-fetal

*PPCM* peripartum cardiomyopathy, *BMI* body mass index, *DCM* dilated cardiomyopathy

**Table 2:** Treatment details of 3 cases

	Case 1	Case 2	Case 3
Onset (postpartum)	PD 7	PD 25	PD 4
Bromocriptine	〇	〇	〇
MCS	IABP	IABPs VA-ECMO Temporary-BiVAD	IABP VA-ECMO LVvent LVAD
IABP, VA-ECMO implantation after onset	3 day (PD 10)	19 days (PD 44)	3 days (PD 7)
VAD implantation after onset	–	20 days (PD 45)	125 days (PD 129)
MCS outcome	Withdrawal	Withdrawal	Withdrawal
MCS implant duration (MCS withdrawal day)	20 days (PD 30)	41 days (PD 85)	286 days (PD 386)
Outcome	Survival	Survival	Survival

*MCS* mechanical circulatory support, *IABP* intra-aortic balloon pump, *VA-ECMO* veno-arterial extracorporeal membrane oxygenation, *BiVAD* biventricular assist device, *VA-ECMO* veno-arterial extracorporeal membrane oxygenation, *LVAD* left ventricular assist device, *PD* postpartum day

**Table 3:** Pre- and post-MCS cardiac function

	Case 1	Case 2	Case 3
MCS	IABP	IABPs VA-ECMO Temporary-BiVAD	IABP VA-ECMO LVvent LVAD
INTERMACS before MCS and symptoms	Profile 2	Profile 1	Profile1
Pre-MCS LVEDd (mm)	63.2	67	69
Pre-MCS LVEF (%)	19.4	22	22
Pre-MCS CO (L/min)	–	3.26	3.47 on PCPS, IABP
Pre-MCS PAP (s/d/m: mmHg)	16/10 (13)	49/20 (27)	43/24 (32) on PCPS, IABP
Pre-MCS LVEDd (mm)	65	46	
Post-MCS LVEF (%)	23	32	56
Post-MCS CO (L/min)	3.9	4.52	4.36
Pre-MCS PAP (s/d/m: mmHg)	21/6 (13)	21/11 (15)	35/15 (24)

*LVEF* left ventricular ejection fraction, *LVEDd* left ventricular end-diastolic diameter, *CO* cardiac output, *PAP* pulmonary atrial pressure, *MCS* mechanical circulatory support, *PCPS* percutaneous cardiopulmonary support, *IABP* intra-aortic balloon pump, *VA-ECMO* veno-arterial extracorporeal membrane oxygenation, *BiVAD* biventricular assist device, *LVAD* left ventricular assist device

### Case 1

A 33-year-old woman (G1P1; height, 156 cm; weight, 46 kg) without a significant medical history developed malaise on postpartum day 7 after normal spontaneous delivery. Her symptom was dyspnea and felling chill when she was referred to cardiology on postpartum day 10. At the time of the cardiology, BNP 930 pg/ml, echocardiography showed LVEF 24%, LVDd/Ds 63/58 mm, moderate mitral regurgitation, blood samples showed mildly elevated liver enzymes and metabolic acidosis. Acute heart failure associated with PPCM was suspected. Myocardial biopsy and coronary angiography were performed, and coronary artery stenosis was excluded. Inotropes, diuretics, and an IABP were inserted on the same day (INTERMAX profile 2). Her hemodynamics was stabilized, but his heart failure worsened with fever, and he was transported to our hospital for LVAD induction on postpartum day 16. At our hospital, with the use of continuous hemodiafiltration, inotropes, and anti-prolactin drugs, the cardiac function LVEF recovered to 30%, CO 2.3 L/min. IABP was withdrawn postpartum day 30. The patient was discharged from the intensive care unit with medical treatment.

### Case 2

A 44-year-old woman (G5P5; height, 151 cm; weight, 56 kg) with left bundle branch block developed acute heart failure and acute pulmonary edema postpartum day 25 after normal vaginal delivery of her fifth child. She was diagnosed PPCM and medical treatment was started with catecholamines, diuretics, and anti-prolactin drugs and fluid restriction. The patient was hemodynamically stabilized with diuretics and inotropes. Postpartum day 43, her symptoms worsened due to non-infectious fever and developed cardiac shock with LVEF of 22% and LVEDd of 67 mm and CO 2.16 L/min. Postpartum day 44, IABP was induced, but the shock status unchanged, PCPS was introduced and transported to our hospital. IABP and VA-ECMO were started. Based on the indication for cardiac transplantation (INTERMAX profile 1) and marked pulmonary edema, the extracorporeal biventricular assist device was inserted postpartum day 45. Subsequently, LVEF improved to 40% and CO 3.47 L/min; therefore, the right ventricular assist device was withdrawn postpartum day 71 and cardiac resynchronization therapy defibrillator (CRT-D) with biventricular pacing was initiated for residual heart failure. Postpartum day 85, the left ventricular assist device (LVAD) was withdrawn, and the patient was discharged from the intensive care unit.

### Case 3

A 37-year-old woman (G1P1; height, 158 cm; weight, 78 kg) without a significant medical history was referred to our hospital with a diagnosis of PPCM. Postpartum day 4, she developed dyspnea with lower leg edema, shock, and LVEF was 33% (INTERMAX profile 1). Medical treatment was started with inotropes and diuretics for heart failure. Subsequently, LVEF reduced to 20%, LVEDd was 69 mm, and mitral regurgitation worsened. IABP, VA-ECMO, and anti-prolactin therapy were started on postpartum day 7. However, congestion progressed with dilation of the left ventricle and left ventricular vent was inserted on postpartum day 11, which resulted in improvement of congestion. Postpartum day 19, VA-ECMO was stopped due to thrombus in its circulation, and at this time the LVEF was 35% and CO 4.76 L/min. Postpartum day 23, we had no choice but to removed IABP and continued medical treatment. However, the cardiac dysfunction could not be controlled and the patient needed IABP again, postpartum day 118. Postpartum day 129, the implantable LVAD was implanted and mitral valve replacement was performed. Postpartum day 386, LVEF recovered to 55% and CO 4.36 L/min, and the LVAD was withdrawn and the patient was discharged from the hospital.

## Discussion

The diagnosis of PPCM is based on the following Demakis criteria: new symptoms of heart failure 1 month before and within 5 months after delivery; no history of heart disease; no other causes of heart failure; and positive echocardiographic findings (LVEF < 45%, fractional shortening of left ventricular diameter, and FS < 30%) [2]. The cause of PPCM remains unknown; however, there are several theories, such as genetic predisposition, viral infections, and inhibition of angiogenesis and cardiotoxicity by truncated prolactin [2]. The risk factors of PPCM include multiple births, age > 30 years, eclampsia, obesity, and complicated gestational hypertension [1–3]. All patients in this report were over 30 years of age. PPCM is relatively rare in Japan but accounts for 3.9% of all maternal deaths [1, 7]. Fortunately, cardiac function is expected to improve in PPCM compared to that in ischemic heart disease, and approximately half of patients with PPCM are expected to recover within 6 months [2]. Poor prognostic factors for PPCM include LVEF < 30% and LVEDd > 60 mm; they indicate a reduced rate of improvement in left ventricular function and an increased risk of invasive treatments, transplantation, and death [8]. All three patients who required MCS in this study had poor prognostic factors.

The first step in the treatment of PPCM is to treat it as general heart failure and arrhythmia. Additionally, prolactin inhibitor has been demonstrated to be effective. Prolactin inhibitor therapy was administered in all three cases in this study. Additionally, PPCM requires concomitant anticoagulation therapy due to the high incidence of thrombosis [9].

Although there are no clear criteria for inducing MCS for PPCM, NYHA III or IV, increased diuretic requirements, dependence on circulatory agonists, organ failure due to poor perfusion, and LVEF < 30% are considered to use MCS to stabilize their general condition [4]. INTERMACS is a US registry acquiring data on patients supported with FDA-approved MCS devices. Within INTERMACS, patients are classified by their signs and symptoms into 7 clinical profiles (1 is most severe and 7 is less severe, Table 4), and the INTERMACS profiles provide guidance for the optimal timing of MCS implantation [10]. Basically, profile 1 cases are indicated for extracorporeal LVADs, and in profile 2, 3 cases are indicated for implantable LVADs [10]. Temporary circulatory procedures such as IABP, ECMO, and Impella are indicated in INTERMACS profiles 1, 2, and 3 [10]. Profiles 6 or 7 patients are, in general, considered too well for MCS.

**Table 4:** INTERMACS clinical profile [4]

Level	Description	Hemodynamic status
1	Critical cardiogenic shock, “crash and burn”	Persistent hypotension despite rapidly escalating inotropic support and eventually IABP, and critical organ hypoperfusion
2	Progressive decline on inotropic support, “sliding on inotropes”	Intravenous inotropic support with acceptable values of blood pressure and continuing deterioration in nutrition, renal function, or fluid retention
3	Stable but inotrope dependent, “dependent stability”	Stability reached with mild to moderate doses of inotropes but demonstrating failure to wean from them because of hypotension, worsening symptoms, or progressive renal dysfunction
4	Resting symptoms, “frequent flyer”	Possible weaning of inotropes but experiencing recurrent relapses, usually fluid retention
5	Exertion intolerant, housebound	Severe limited tolerance for activity, comfortable at rest with some volume overload and often with some renal dysfunction
6	Exertion limited, “walking wounded”	Less severe limited tolerance for activity and lack of volume overload, fatigue easily
7	Advanced NYHA III “symptoms, placeholder”	Patient without current or recent unstable fluid balance, NYHA class II or III

*INTERMACS* Interagency Registry for Mechanically Assisted Circulatory Support, *NYHA* New York Heart Association functional classification

In this reports, one patient was INTERMACS profile 2 and other were 1 at the time of MCS induction, and the profile 2 case induced IABP at the same day of cardiology visit before heart failure progress.

Traditionally, durable MCSs were reserved primarily for patients with end-stage heart failure who were deemed candidates for transplantation as “bridge to transplantation” and long-term destination therapy for non-transplant candidates in Japan and other countries with a shortage of transplant donors [11, 12]. In contrast, VAD may be implanted for recovery of own heart function; subsequent withdrawal of VAD is called “bridge to recovery (BTR)” [13–17]. By reducing the hemodynamic pre-load and resting the myocardium using an implanted VAD, the original cardiac function is restored and the VAD can be withdrawn. There are a few case reports of successful BTR using the LVAD in PPCM [5, 6]. Especially regarding to non-ischemic cardiomyopathy, a characteristic of patients with successful BTR is the early introduction to VAD support [18]. In the BTR report on non-ischemic myocardium, 73% of patients who had an LVAD implanted within the first 4 months of heart failure symptoms recovered, while no recovery was seen in subjects who had more than 4 months [18]. In this report, two cases requiring LVADs. In our case 3, IABP was introduced early, but it took a long time to introduce LVAD. The patient eventually recovered, but it took longer time for treatment than case 2 which induced LAVD within 4 months of onset, at 18 days after onset. Our observations suggest the possibility of improving recovery and prognosis in PPCM using early MCSs. Although the invasive nature of the LVAD surgery may raise the hurdle, the introduction of MCS for PPCM that does not respond to medical therapy should be done aggressively. PPCM that does not respond to medical therapy should be referred to a center with expertise in the management of MCS and patients with advanced PPCM heart failure.

## Conclusion

We presented three cases of severe PPCM that required MCS. All the patients survived and resumed their social lives.

## Data Availability

Please contact the corresponding author for data requests.

## Abbreviations

PPCM: Peripartum cardiomyopathy
MCS: Mechanical circulatory support
IABP: Intra-aortic balloon pump
PCPS: Percutaneous caidiopulmonary support
VA-ECMO: Veno-arterial extra-corporeal membrane oxygenation
BiVAD: Biventricular assist device
LVEF: Left ventricular ejection fraction
LVAD: Left ventricular assist device
VAD: Ventricular assist devices
LVEDd: Left ventricular end-diastolic diameter
CRT-D: Cardiac resynchronization therapy defibrillator
BTR: Bridge to recover
NYHA: New York Heart Association functional classification
